# Brain‐targeted Nano‐ERASER downregulates PD‐L1 for Alzheimer’s disease therapy

**DOI:** 10.1002/alz70859_102249

**Published:** 2025-12-25

**Authors:** Peisheng Xu

**Affiliations:** ^1^ University of South Carolina, Columbia, SC USA

## Abstract

**Background:**

Programmed death ligand 1 (PD‐L1) plays a pivotal role in modulating neuroinflammation in Alzheimer’s disease (AD), and PD‐L1 upregulation is widely observed in the brains of AD patients. This dysregulation hinders Aβ plaque clearance and leads to detrimental microglial activation.

**Method:**

To regulate the PD‐L1 in the brain, we developed a brain‐targeted Nano‐ERASER system (BTN‐PDL1).

**Result:**

BTN‐PDL1 can effectively cross the BBB, deplete PD‐L1 through a Trim21‐mediated proteasomal degradation in microglia and astrocytes, and revive their functions, which can subsequently clear toxic Aβ fibrils and attenuate neuroinflammation. Animal behavior assay revealed that BTN‐PDL1 stopped the progression of AD and improved learning and cognitive capacity in 5XFAD mouse model.

**Conclusion:**

Since protein malfunction and neuroinflammation prevail in many CNS disease conditions, the success of BTN‐PDL1 could also be beneficial for the treatment of AD, Parkinson's disease, Huntington’s disease, Amyotrophic lateral sclerosis, stroke, and traumatic brain injury.

